# Efficacy of heel lifts for lower limb musculoskeletal conditions: A systematic review

**DOI:** 10.1002/jfa2.12031

**Published:** 2024-06-15

**Authors:** Jaryd Bourke, Shannon Munteanu, Eman Merza, Alessandro Garofolini, Simon Taylor, Peter Malliaras

**Affiliations:** ^1^ Physiotherapy Department School of Primary and Allied Health Care Faculty of Medicine Nursing and Health Science Monash University Clayton Victoria Australia; ^2^ Discipline of Podiatry School of Allied Health Human Services and Sport La Trobe University Melbourne Victoria Australia; ^3^ Institute for Health and Sport (IHES) Victoria University Melbourne Victoria Australia

**Keywords:** musculoskeletal pain, orthotic devices, podiatry, rehabilitation, systematic review

## Abstract

**Introduction:**

The objective of this systematic review is to determine the benefits and harms of heel lifts to any comparator for lower limb musculoskeletal conditions.

**Methods:**

Ovid MEDLINE, Ovid AMED, Ovid EMCARE, CINAHL Plus and SPORTDiscus were searched from inception to the end of May 2024. Randomised, quasi‐randomised or non‐randomised trials comparing heel lifts to any other intervention or no‐treatment were eligible for inclusion. Data was extracted for the outcomes of pain, disability/function, participation, participant rating of overall condition, quality of life, composite measures and adverse events. Two authors independently assessed risk of bias and certainty of evidence using the GRADE approach at the primary time point 12 weeks (or next closest).

**Results:**

Eight trials (*n* = 903), investigating mid‐portion Achilles tendinopathy, calcaneal apophysitis and plantar heel pain were included. Heel lifts were compared to exercise, ultrasound, cryotherapy orthotics, stretching, footwear, activity modification, felt pads and analgesic medication. No outcome was at low risk of bias and few effects (2 out of 47) were clinically important. Low‐certainty evidence (1 trial, *n* = 199) indicates improved pain relief (55.7 points [95% CI: 50.3–61.1], on a 100 mm visual analogue scale) with custom orthotics compared to heel lifts at 12 weeks for calcaneal apophysitis. Very low‐certainty evidence (1 trial, *n* = 62) indicates improved pain and function with heel lifts over indomethacin (35.5 points [95% CI: 21.1–49.9], Foot Function Index) at 12 months for plantar heel pain.

**Conclusions:**

Few trials have assessed the benefits and harms of heel lifts for lower limb musculoskeletal conditions. Only two outcomes out of 47 showed clinically meaningful between group differences. However, due to very low to low certainty evidence we are unable to be confident in the results and the true effect may be substantially different.

**Registration:**

PROSPERO registration number CRD42022309644.

## 1 INTRODUCTION

Non‐traumatic lower limb musculoskeletal conditions are common, reported to have an incidence of 19%–79% in the running population [1] and 16% in the sedentary population [2]. People with these lower limb conditions often report localised load‐related pain and disability, which affects sporting performance and promotes sedentary behaviors, with up to one third of all injured reported to not returning to their previous activity levels [2]. Considering that physical inactivity is a risk factor for multisystem disease [3], mental illness [4] and morbidity [5], injury to the lower limb can have a greater impact on individuals beyond sporting inconvenience.

First‐line treatments for non‐traumatic lower limb musculoskeletal conditions usually involve graduated exercise programs coupled with advice about managing occupational and sporting loads [6, 7, 8]; however, it is accepted that these treatments are not always successful and other interventions are sometimes needed [9]. One such option is heel lifts, which are shoe inserts designed to plantarflex the foot at the ankle joint [10]. Expert narratives and clinical guidelines support their use for a range of lower limb musculoskeletal conditions including, but not limited to, plantar heel pain [11], Achilles tendinopathy [12], calcaneal apophysitis [13] and anterior ankle impingement [14]. However, at present, there are no empirically proven guidelines to inform the recommended material (e.g., cork, polyurethane, EVA, etc.) or height of heel lift to use.

The speculated mechanisms by which heel lifts exert their effect are diverse and are yet to be fully understood [15]. Nonetheless, any clinically observed improvements in pain and function with heel lifts are often associated with alterations in various biomechanical variables, including temporospatial parameters [16], kinematics [17], dynamic plantar pressures [18], kinetics (e.g., joint moments [19]) and muscle function [20]. It is believed that these changes form the basis for the therapeutic effects of heel lifts. However, the biomechanical rationale for their application remains uncertain due to the limited and inconclusive evidence available in this field [15]. Despite the wide availability and use of heel lifts, no study has systematically reviewed research investigating the efficacy of heel lifts for lower limb musculoskeletal conditions. Summarising the musculoskeletal conditions that benefit or are at harm from heel lifts would serve as a valuable resource for clinicians to aid them in their decision‐making process.

### 1.1 Objectives

To determine the benefits and harms of heel lifts for lower limb musculoskeletal conditions compared to another intervention or a no treatment control (placebo, sham or wait‐and‐see).

## 2 METHODS

This systematic review was developed and reported according to the Preferred Reporting of Systematic Reviews and Meta‐Analysis (PRISMA) guidelines [21]. This systematic review was prospectively registered in the PROSPERO database; registration: CRD42022309644.

## 3 DEVIATIONS FROM STUDY REGISTRATION AND THE STUDY PROTOCOL

In our study registration, we also planned an analysis of biomechanical studies. Following peer‐review, we removed the biomechanical analysis, as it did not add anything to this systematic review. Instead, we have focussed on a high‐quality review of clinical outcomes.

## 4 CRITERIA FOR CONSIDERING STUDIES FOR THIS REVIEW

### 4.1 Types of studies

Randomised, quasi‐randomised and non‐randomised trials were included if one study arm used heel lifts (as an adjunct or primary intervention) compared to another intervention or a control (e.g., placebo, sham or wait‐and‐see) [22]. Trials that were unpublished, not peer reviewed or non‐English written studies were excluded.

### 4.2 Types of participants

We included all trials that recruited participants with a musculoskeletal condition, which may include but is not limited to conditions such as, Achilles tendinopathy and plantar heel pain. There were no restrictions on age or sex/gender. Trials including participants with neurological disorders, limb length discrepancies or a history of amputation were excluded.

### 4.3 Types of interventions

Heel lifts were defined as being removable (attached to the participant's barefoot or in the shoe) or a feature in‐built into a shoe intended to plantarflex the foot at the ankle joint [15]. Any comparisons were permitted and could include no treatment (placebo, sham or wait‐and‐see) or any intervention such as orthotics, exercise and education.

### 4.4 Types of outcome measures

Primary outcomes were pain, disability/function, participation, participant rating of overall condition, quality of life and composite measures, as they are recommended in the consensus statement for tendinopathy outcomes [23]. The number of participants reporting any adverse events (secondary outcome) were also extracted to provide a balanced perspective of harms.

### 4.5 Timing of outcome measures

The primary time point was 12 weeks (or the next closest time point) as it has commonly been used as a primary endpoint in clinical trials of interventions for musculoskeletal conditions [24, 25]. Outcome measures were obtained for the following time points: short term (0–6 weeks), medium term (>6–12 weeks), long term (>12 weeks) to comprehensively evaluate the benefits and harms of the interventions [26]. If two follow‐up assessments were reported within one of the defined time points, the results of the latter of the two assessments were selected [27].

## 5 SEARCH METHODS FOR IDENTIFICATION OF STUDIES

Database searching was performed across Ovid MEDLINE, Ovid AMED, Ovid EMCARE, CINAHL Plus and SPORTDiscus platforms from inception to the end of May 2024. The search strategy used a combination of key words pertinent to the research questions. The search syntax and related number of items found with each database can be found in Supporting Information S1. Forward and backward searches were conducted to identify other eligible trials (forward searches in Google Scholar and PubMed).

## 6 DATA COLLECTION AND ANALYSIS

### 6.1 Selection of studies

All trials identified from the search were downloaded into Endnote X9 (Thomson Reuters, Philadelphia, PA) and duplicates deleted by a single author (JB). Titles and abstracts of the trials were screened independently by two authors for inclusion (JB, EM) and any discrepancies were resolved by a third author (PM). If further information was required, the full‐text was obtained.

### 6.2 Data extraction and management

Relevant data were extracted and mapped to a characteristics table independently by two authors (JB, EM) and any discrepancies were resolved by a third author (PM). Additional data was retrieved from published protocols and trial registrations, where available. Any missing data was requested from the corresponding authors by a single author (JB) and considered unsuccessful if there was no reply after two attempts. The following was extracted from eligible trials:

- trial characteristics: sample size, first author name, year of publication, type of trial (e.g., parallel, cross‐over);
- participant characteristics: age, sex/gender, activity levels, adherence to the heel lifts, type of injury, duration of symptoms, type of footwear;
- heel lift and comparator characteristics: height and material of the heel lifts and a description of the comparator intervention(s);
- summary data for each outcome: number of events and number of participants per group for dichotomous outcomes, mean and standard deviation per group for continuous outcomes.

Definitions for the outcomes and our a *priori* decision rules for extracting data from multiple reported outcomes in trials can be found in the Supporting Information S2.

### 6.3 Assessment of risk of bias in included studies

Risk of bias assessment was performed using the revised Cochrane Collaboration tool for assessing risk of bias (RoB 2.0) [28, 29] independently by two authors (JB and SM). Disagreements were resolved by a third author (PM). An outcome was considered to have a high risk of bias if at least one of the criteria was rated high risk [30]. To be considered low risk of bias, all criteria had to be rated low risk [30]. Any outcomes not meeting these criteria were considered to be at some concern of risk of bias [30].

### 6.4 Measure of treatment effect

Measures of treatment effect were calculated as specified in the Cochrane Handbook for Systematic Reviews of Interventions [31]. For dichotomous outcomes, estimates were analyzed as risk ratios (RRs) with 95% confidence intervals (CIs). For continuous outcomes, estimates were analyzed as mean differences (MDs) with 95% CIs. We assumed a relative risk difference of 25% was a minimal clinically important difference (MCID) for dichotomous outcomes. Many different continuous outcome measures were included in this review; for example, pain was assessed using the visual analogue scale, subscale of the Foot Function Index, Faces Pain Scale and a 5‐point scale. Our assumed MCID for the different outcome measures is listed in Table 1. If we were unable to identify a suitable MCID, we used 10% of the maximum possible score of the outcome.

Two trials [36, 37] described their results in median and interquartile ranges; to calculate the MDs, the median was assumed as the mean and the interquartile range divided by 1.35 to identify the approximate standard deviations [38]. Where calculating MDs was not possible, a quote extracted from that trial was presented descriptively.

**TABLE 1:** Minimal clinically important difference for the outcomes included in this review.

Outcome	MCID
Visual analogue scale	8 mm [32]
VISA‐A	14 points [33]
FFI	12 points [34]
Face pain scale—Revised	2 points [35]

### 6.5 Assessment of the certainty of the evidence

Assessment of the certainty at the outcome level was undertaken using the GRADE approach for the primary time point (12 weeks) [39]. Two authors (JB, EM) independently assessed the quality of evidence, with a third author available to resolve any discrepancies (PM). The certainty of evidence for each outcome was graded as high, moderate, low or very low and presented in a Summary of Findings table [40]. We justified all decisions to downgrade the certainty of evidence using footnotes and made comments to aid the reader's understanding of the review where necessary. Our rules for determining the GRADE judgment for each outcome can be found in the Supporting Information S3, which were derived from the GRADE handbook [39] and consensus among authors (JB, PM, SM).

### 6.6 Data synthesis

Meta‐analysis was planned for trials with similar characteristics (e.g., participants, interventions, outcomes). Data was categorised according to the condition (e.g., plantar heel pain, Achilles tendinopathy, etc.). Different labels of conditions were grouped under a recommended label; for example, plantar fasciitis and ‘heel spur syndrome’ were grouped as plantar heel pain [11]. All MD calculations and quotes extracted from the trials can be found in Supporting Information S4.

## 7 RESULTS

### 7.1 Trial selection

**FIGURE 1:**
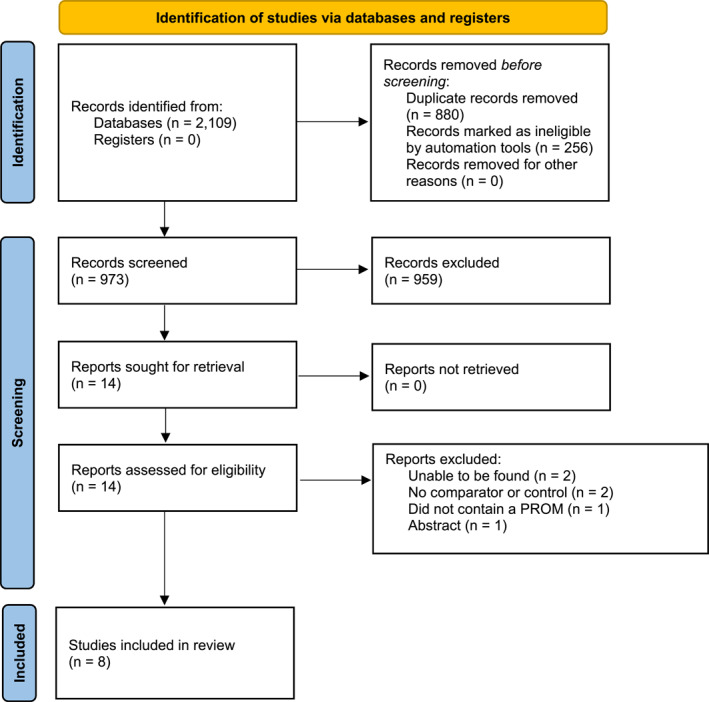
Flow of studies through the review process.

Initially, 2109 records were retrieved and 880 deleted as duplicates and 256 removed by an automation tool to remove any records unrelated to humans. Of these, we assessed 14 in full‐text and excluded 6 after full‐text evaluation [41, 42, 43, 44, 45, 46], which yielded 8 [36, 37, 47, 48, 49, 50, 51, 52] trials eligible for inclusion (Figure 1).

### 7.2 Trial characteristics

The characteristics of the included trials are shown in Supporting Information S4. All trials were randomised trials, seven were classified as parallel group superiority trials [36, 37, 47, 48, 50, 51, 52] and one used a 2 × 2 factorial design [49]. A total of 903 participants were included and sample sizes ranged from 23 to 208. The duration of the trials ranged from 8 to 52 weeks. Overall, participants were typically young adults (mean age = 31 years) and more than half were male (52%). The median height of heel lifts where reported was 8 mm (average = 9 mm, ranging from 6 to 12 mm) and they were manufactured from a variety of materials (e.g., ethyl vinyl acetate, silicone, proprietary products). The musculoskeletal conditions and interventions assessed were all single trial comparisons and were as follows.

#### 7.2.1 Mid‐portion Achilles tendinopathy

- heel lifts and activity modification versus eccentric calf exercises and activity modification [36];
- heel lifts, therapeutic ultrasound, stretching and strengthening exercises for ‘posterior leg structures’ and activity modification versus therapeutic ultrasound, stretching and strengthening exercises for ‘posterior leg structures’ and activity modification [50].

#### 7.2.2 Calcaneal apophysitis

- heel lifts, cryotherapy, calf stretching and activity modification versus custom orthotics, cryotherapy, calf stretching and activity modification [47];
- heel lifts, calf stretching and cryotherapy versus prefabricated orthotics, calf stretching and cryotherapy [49];
- heel lifts, calf stretching and cryotherapy versus prefabricated orthotics, new Adidas runners, calf stretching and cryotherapy [49];
- heel lifts versus activity modification [52];
- heel lifts versus eccentric calf exercise [52].

#### 7.2.3 Plantar heel pain

- heel lifts, anti‐inflammatory medication and plantar fascia stretching versus custom orthotics, anti‐inflammatory medication and plantar fascia stretching [37];
- heel lifts and a heat pack versus 75 mg of indomethacin [48].
- heel lifts and a heat pack versus plantar fascia stretching and sham calf stretching [48];
- heel lifts and a heat pack versus calf stretching and sham plantar fascia stretching [48];
- heel lifts and ‘Achilles’ and plantar fascia stretching versus felt pads and ‘Achilles’ and plantar fascia stretching [51];
- heel lifts and ‘Achilles’ and plantar fascia stretching versus custom orthotics and ‘Achilles’ and plantar fascia stretching [51];
- heel lifts and ‘Achilles’ and plantar fascia stretching versus ‘Achilles’ and plantar fascia stretching [51].

### 7.3 Risk of bias assessment

**FIGURE 2:**
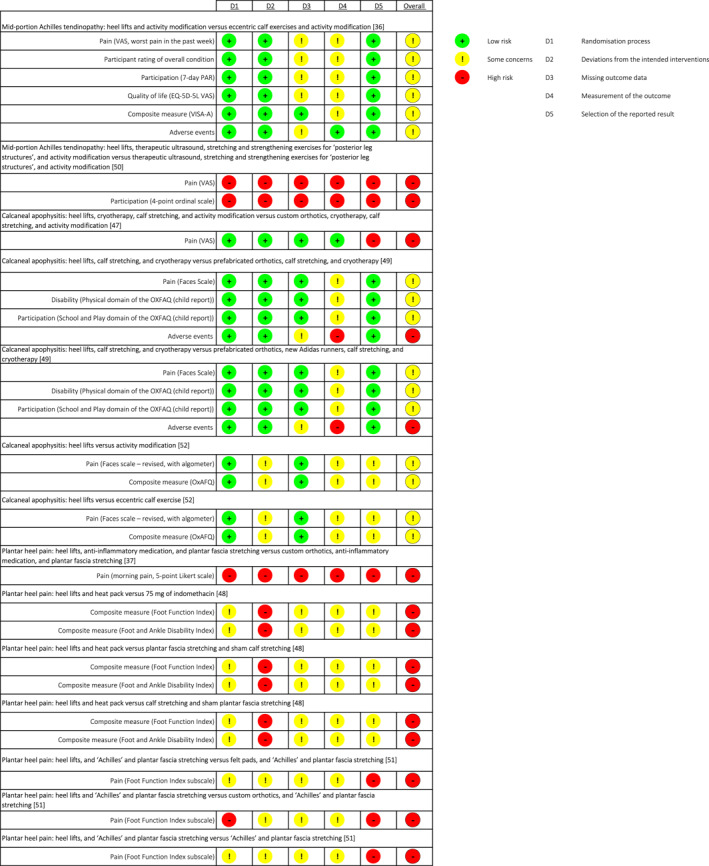
Risk of bias summary for each included outcome at the primary time point.

The risk of bias for each included outcome is summarised in Figure 2. No outcome was judged to be at low risk of bias. Half of the outcomes were judged to have some concern of risk of bias (16 out of 31) and the other half at high risk of bias (15 out of 31). Most of the outcomes (94%) were downgraded due to being unable to blind the investigator, participant or both. Our rationale for the judgements can be found in the Supporting Information S5.

### 7.4 Effects of intervention

**FIGURE 3:**
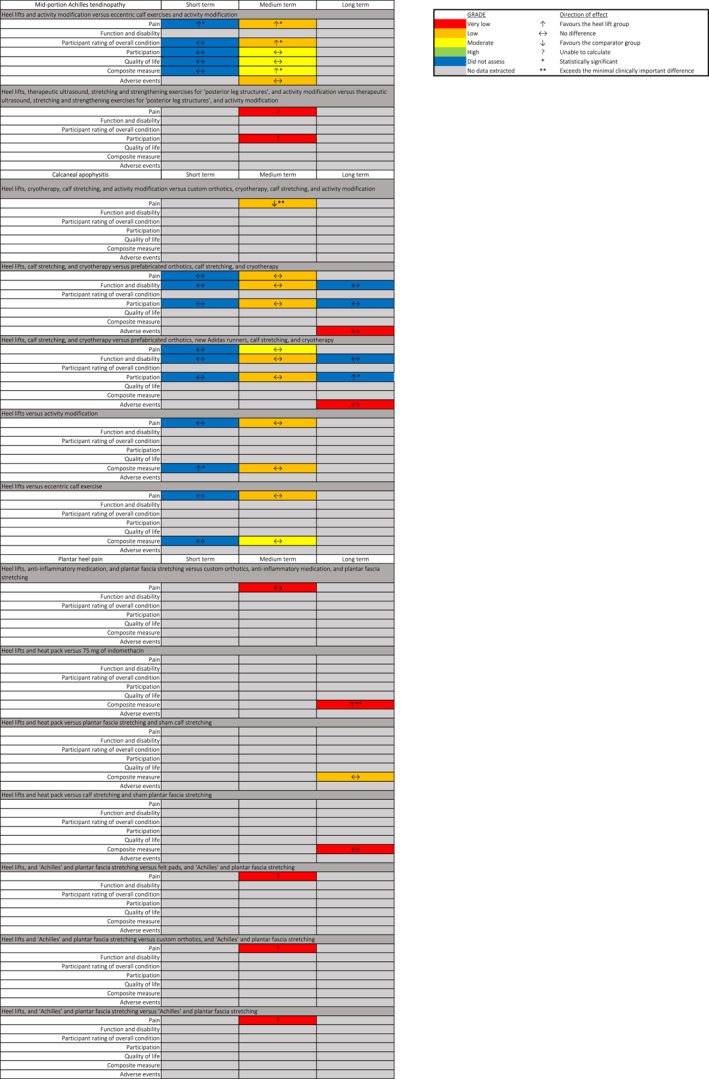
Summary of outcomes for all time points.

Results for the primary time point (12 weeks or next closest) are shown in the Summary of Findings table (Table 2) and for all time points in Figure 3. The full description of the interventions, outcomes assessed and time points extracted is located in Supporting Information S4. Meta‐analysis was not possible as there was significant heterogeneity in the comparator interventions between trials (no trial used the same heel lift and comparison). Instead, the findings were presented descriptively.

**TABLE 2:** Summary of findings table for the primary time point (12 weeks).

Outcomes	Mean difference (95% CI)	No of participants (studies)	Certainty of the evidence (GRADE)	Comments
Mid‐portion Achilles tendinopathy: Heel lifts and activity modification versus eccentric calf exercises and activity modification [36]				
Pain (VAS, worst pain in the past week)	−19.5 (−31.71 to −7.29)^a^	80 (1 RCT)	⨁⨁◯◯ Low	Downgraded due to some concerns of risk of bias and group size < OIS.
Participant/patient rating overall condition	Relative risk: 1.41 (1.02–1.95)^a^	80 (1 RCT)	⨁⨁◯◯ Low	Downgraded due to some concerns of risk of bias and group size < OIS.
Participation (7‐day PAR)	−51.5 (−512.88–409.88)	80 (1 RCT)	⨁⨁⨁◯ Moderate	Downgraded due to some concerns of risk of bias.
Quality of life (EQ‐5D‐5 L VAS)	2.1 (−3.39–7.59)	80 (1 RCT)	⨁⨁⨁◯ Moderate	Downgraded due to some concerns of risk of bias.
Composite measure (VISA‐A)	12.3 (3.52–21.08)^a^	80 (1 RCT)	⨁⨁⨁◯ Moderate	Downgraded due to some concerns of risk of bias.
Adverse events	Relative risk: 1.05 (0.68–1.61)	80 (1 RCT)	⨁⨁◯◯ Low	Downgraded due to high risk of bias and group size < OIS.
Mid‐portion Achilles tendinopathy: Heel lifts, therapeutic ultrasound, stretching and strengthening exercises for ‘posterior leg structures’ and activity modification versus therapeutic ultrasound, stretching and strengthening exercises for ‘posterior leg structures’ and activity modification [50]				
Pain (VAS)	n/a	33 (1 RCT)	⨁◯◯◯ Very low	Downgraded due to high risk of bias, unable to calculate OIS and no description of how the condition was diagnosed.
Participation (4‐point ordinal scale)	n/a	33 (1 RCT)	⨁◯◯◯ Very low	Downgraded due to high risk of bias, unable to calculate OIS and no description of how the condition was diagnosed.
Calcaneal apophysitis: Heel lifts, cryotherapy, calf stretching and activity modification versus custom orthotics, cryotherapy, calf stretching and activity modification [47]				
Pain (VAS)	55.7 (50.27–61.13)^b^	199 (1 RCT)	⨁⨁◯◯ Low	Downgraded due to a high risk of bias.
Calcaneal apophysitis: Heel lifts, calf stretching and cryotherapy versus prefabricated orthotics, calf stretching and cryotherapy [49]				
Pain (Faces scale)	0 (−0.57 to 0.57)	61 (1 RCT)	⨁⨁◯◯ Low	Downgraded due to some concerns of risk of bias and group size < OIS.
Disability (physical domain of the OXFAQ (child report))	−0.49 (−10.68–9.70)	61 (1 RCT)	⨁⨁◯◯ Low	Downgraded due to some concerns of risk of bias and group size < OIS.
Participation (school and play domain of the OXFAQ (child report))	3.32 (−3.75–10.39)	61 (1 RCT)	⨁⨁◯◯ Low	Downgraded due to some concerns of risk of bias and group size < OIS.
Adverse events	Relative risk: 0 (n/a)	61 (1 RCT)	⨁◯◯◯ Very low	Downgraded due to high risk of bias and group size < OIS.
Calcaneal apophysitis: Heel lifts, calf stretching and cryotherapy versus prefabricated orthotics, new Adidas runners, calf stretching and cryotherapy [49]				
Pain (faces scale)	0.06 (−0.45–0.57)	60 (1 RCT)	⨁⨁⨁◯ Moderate	Downgraded due to some concerns of risk of bias.
Disability (physical domain of the OXFAQ (child report))	−4.96 (−13.90–3.98)	60 (1 RCT)	⨁⨁◯◯ Low	Downgraded due to some concerns of risk of bias and group size < OIS.
Participation (school and play domain of the OXFAQ (child report))	−2.92 (−8.60–2.76)	60 (1 RCT)	⨁⨁◯◯ Low	Downgraded due to some concerns of risk of bias and group size < OIS.
Adverse events	Relative risk: 0 (n/a)	60 (1 RCT)	⨁◯◯◯ Very low	Downgraded due to high risk of bias and group size < OIS.
Calcaneal apophysitis: Heel lifts versus activity modification [52]				
Pain (faces scale—revised, with algometer)	0.4 (−1.01–1.81)	63 (1 RCT)	⨁⨁◯◯ Low	Downgraded due to some concerns of risk of bias and compressing apophysis with algometer not reflective of clinical practice.
Composite measure (OXFAQ)	4.8 (−0.11–9.71)	63 (1 RCT)	⨁⨁◯◯ Low	Downgraded due to some concerns of risk of bias and having a wide confidence interval.
Calcaneal apophysitis: Heel lifts versus eccentric calf exercise [52]				
Pain (faces scale—revised, with algometer)	0.5 (−0.95–1.95)	65 (1 RCT)	⨁⨁◯◯ Low	Downgraded due to some concerns of risk of bias and compressing apophysis with algometer not reflective of clinical practice.
Composite measure (OXFAQ)	3 (−1.22–7.22)	65 (1 RCT)	⨁⨁⨁◯ Moderate	Downgraded due to some concerns of risk of bias.
Plantar heel pain: Heel lifts, anti‐inflammatory medication and plantar fascia stretching versus custom orthotics, anti‐inflammatory medication and plantar fascia stretching [37]				
Pain (morning pain, 5‐point Likert scale)	0.5 (−0.27–1.27)	60 (1 RCT)	⨁◯◯◯ Very low	Downgraded due to high risk of bias and group size < OIS.
Plantar heel pain: Heel lifts and heat pack versus 75 mg of indomethacin [48]				
Composite measure (foot function index)	−35.5 (−49.4 to −21.06)^b^	62 (1 RCT)	⨁◯◯◯ Very low	Downgraded due to high risk of bias and group size < OIS.
Composite measure (foot and ankle disability index)	16.5 (6.56–26.44)^a^	62 (1 RCT)	⨁◯◯◯ Very low	Downgraded due to high risk of bias and group size < OIS.
Plantar heel pain: Heel lifts and heat pack versus plantar fascia stretching and sham calf stretching [48]				
Composite measure (foot function index)	4.9 (−1.7–11.59)	62 (1 RCT)	⨁⨁◯◯ Low	Downgraded due to high risk of bias.
Composite measure (foot and ankle disability index)	−3.2 (−9.97–3.57)	62 (1 RCT)	⨁⨁◯◯ Low	Downgraded due to high risk of bias.
Plantar heel pain: Heel lifts and heat pack versus calf stretching and sham plantar fascia stretching [48]				
Composite measure (foot function index)	−3.8 (−13.22–5.62)	60 (1 RCT)	⨁◯◯◯ Very low	Downgraded due to high risk of bias and having a wide confidence interval.
Composite measure (foot and ankle disability index)	4.2 (−3.90–12.30)	60 (1 RCT)	⨁◯◯◯ Very low	Downgraded due to high risk of bias and having a wide confidence interval.
Plantar heel pain: Heel lifts and ‘Achilles’ and plantar fascia stretching versus felt pads and ‘Achilles’ and plantar fascia stretching [51]				
Pain (foot function index subscale)	n/a	98 (1 RCT)	⨁◯◯◯ Very low	Downgraded due to high risk of bias, unable to calculate OIS and ‘felt pad’ not reflective of clinical practice.
Plantar heel pain: Heel lifts and ‘Achilles’ and plantar fascia stretching versus custom orthotics and ‘Achilles’ and plantar fascia stretching [51]				
Pain (foot function index subscale)	n/a	93 (1 RCT)	⨁◯◯◯ Very low	Downgraded due to high risk of bias and unable to calculate OIS.
Plantar heel pain: Heel lifts and ‘Achilles’ and plantar fascia stretching versus ‘Achilles’ and plantar fascia stretching [51]				
Pain (foot function index subscale)	n/a	97 (1 RCT)	⨁◯◯◯ Very low	Downgraded due to high risk of bias and unable to calculate OIS.

*Note*: GRADE Working Group grades of evidence. High quality: Further research is very unlikely to change our confidence in the estimate of effect. Moderate quality: Further research is likely to have an important impact on our confidence in the estimate of effect and may change the estimate. Low quality: Further research is very likely to have an important impact on our confidence in the estimate of effect and is likely to change the estimate. Very low quality: We are very uncertain about the estimate.

^a^ Statistically significant.

^b^ Statistically significant and exceeds the MID.

#### 7.4.1 Mid‐portion Achilles tendinopathy

##### 7.4.1.1 Heel lifts and activity modification versus eccentric calf exercises and activity modification [36]

At the primary timepoint (reported at 12 weeks), heel lifts were found to be superior to eccentric calf exercise in reducing pain severity by 19.5 points on a 100 mm visual analogue scale (VAS) (95% CI: 7.29–31.71), the VISA‐A questionnaire by 12.3 points (95% CI: 3.52–21.08) and participant rating of overall condition (relative risk: 1.41, 95% CI: 1.02–1.95) (low to moderate certainty evidence). The rate of adverse events was similar between groups (relative risk: 1.05, 95% CI: 0.71–1.54) and included developing areas of new pain (lower back, hips, knees, feet or ankles) and/or blisters (low certainty evidence). In the short‐term (reported at 6 weeks), heel lifts were found to be superior to eccentric calf exercise in reducing pain severity on a 100 mm VAS outcome by 15.1 points (95% CI: 2.75–27.45).

##### 7.4.1.2 Heel lifts, therapeutic ultrasound, stretching and strengthening exercises for ‘posterior leg structures’ and activity modification versus therapeutic ultrasound, stretching and strengthening exercises for ‘posterior leg structures’ and activity modification [50]

At the primary time point (reported at 8 weeks), mean differences were unable to be calculated. The trial authors reported that the “benefit of viscoelastic pads widely used by athletes was not substantiated” (very low certainty evidence).

#### 7.4.2 Calcaneal apophysitis

##### 7.4.2.1 Heel lifts, cryotherapy, calf stretching and activity modification versus custom orthotics, cryotherapy, calf stretching and activity modification [47]

At the primary timepoint (reported at 12 weeks), heel lifts were found to be inferior to custom orthotics in reducing pain severity by 55.7 points on a 100 mm VAS (95% CI: 50.27–61.13, low certainty evidence).

##### 7.4.2.2 Heel lifts, calf stretching and cryotherapy versus prefabricated orthotics, calf stretching and cryotherapy [49]

At the primary timepoint (reported at 8 weeks), there were no differences between groups for any outcome (low certainty evidence) and no participant reported any adverse reaction to the interventions (very low certainty evidence). In the short‐term (reported at 4 weeks) and long‐term (reported at 52 weeks), there were no differences between groups for any outcome.

##### 7.4.2.3 Heel lifts, calf stretching and cryotherapy versus prefabricated, new Adidas runners, calf stretching and cryotherapy [49]

At the primary timepoint (reported at 8 weeks), there were no differences between groups for any outcome (low certainty evidence) and no participant reported any adverse reaction to the interventions (very low certainty evidence). In the short‐term (reported at 4 weeks), there were no differences between groups for any outcome. In the long‐term (reported at 52 weeks), heel lifts were found to be superior to the prefabricated orthotics and new Adidas runners in the Oxford Foot Ankle Questionnaire—School and Play domain by 6.7 points (95% CI: 0.84–12.56).

##### 7.4.2.4 Heel lifts versus activity modification [52]

At the primary timepoint (reported at 12 weeks), there were no differences between groups for any outcome (low certainty evidence). In the short‐term (reported at 6 weeks), heel lifts were found to be superior to the activity modification group in the Oxford Foot and Ankle Questionnaire by 4.5 points (95% CI: 1.24–7.76).

##### 7.4.2.5 Heel lifts versus eccentric calf exercise [52]

At the primary (reported at 12 weeks), there were no differences between groups for any outcome (low to moderate certainty evidence). In the short‐term (reported at 6 weeks), there were no differences between groups for any outcome.

#### 7.4.3 Plantar heel pain

##### Heel lifts, anti‐inflammatory medication and plantar fascia stretching versus custom orthotics, anti‐inflammatory medication and plantar fascia stretching [37]

At the primary timepoint (reported at 12 weeks), there were no differences between groups (very low certainty evidence).

##### 7.4.3.1 Heel lifts and heat pack versus 75 mg of indomethacin [48]

At the primary timepoint (reported at 52 weeks), heel lifts were found to be superior to 3 weeks of 75 mg of Indomethacin in the Foot Function Index (FFI) by 35.5 points (95% CI: 21.06–49.94) and Foot and Ankle disability Index (FADI) by 16.5 points (95% CI, 6.56–26.44) (very low certainty evidence).

##### 7.4.3.2 Heel lifts and heat pack versus plantar fascia stretching and sham calf stretching [48]

At the primary timepoint (reported at 52 weeks), there were no differences between groups for any outcome (low certainty evidence).

##### 7.4.3.3 Heel lifts and heat pack versus calf stretching and sham plantar fascia stretching [48]

At the primary timepoint (reported at 52 weeks), there were no differences between groups for any outcome (very low certainty evidence).

##### 7.4.3.4 Heel lifts and ‘Achilles’ and plantar fascia stretching versus felt pads and ‘Achilles’ and plantar fascia stretching [51]

At the primary time point (reported at 8 weeks), mean differences were unable to be calculated. The trial authors reported that the silicone heel lift group improved by ‘95%’ compared to the felt pads ‘81%’ (very low certainty evidence).

##### 7.4.3.5 Heel lifts and ‘Achilles’ and plantar fascia stretching versus custom orthotics and ‘Achilles’ and plantar fascia stretching [51]

At the primary time point (reported at 8 weeks), mean differences were unable to be calculated. The trial authors reported that the silicone heel lift group improved by ‘95%’ compared to the custom orthotics ‘68%’ (very low certainty evidence).

##### 7.4.3.6 Heel lifts and ‘Achilles’ and plantar fascia stretching versus ‘Achilles’ and plantar fascia stretching [51]

At the primary time point (reported at 8 weeks), mean differences were unable to be calculated. The trial authors reported that the silicone heel lift group improved by ‘95%’ compared to the stretching group ‘72%’ (very low certainty evidence).

## 8 DISCUSSION

This is the first systematic review to investigate the benefits and harms of heel lifts for lower limb musculoskeletal conditions. Although heel lifts are recommended for numerous lower limb musculoskeletal conditions, such as posterior leg muscle strains [42], the existing evidence is limited to eight trials and three musculoskeletal conditions (mid‐portion Achilles tendinopathy, plantar heel pain and calcaneal apophysitis). Overall, the current evidence indicates that heel lifts may be effective for mid‐portion Achilles tendinopathy [36] and plantar heel pain [48] when compared to eccentric exercise and 75 mg of Indomethacin, respectively; but not for calcaneal apophysitis [47] when compared to custom orthotics. The harms of heel lifts are uncertain.

The benefit of heel lifts over exercise for mid‐portion Achilles tendinopathy in perception of treatment effect, reducing pain severity and improving VISA‐A scores, is a noteworthy finding given that the latest clinical practice guidelines recommend exercise, but they do not mention heel lifts [53]. Heel lifts are inexpensive, widely available and do not have to contend with the same behavioral change demands of a complex intervention such as exercise [54]. However, it is worthwhile noting the between group difference for the trial reporting favourable effects of heel lifts over eccentric calf muscle exercise, did not exceed the threshold of the minimal clinically important difference, which means it is unclear whether patients would be able to discern an appreciable difference if they were prescribed either intervention [55]. The benefit of heel lifts over Indomethacin for plantar heel pain, reflected in the FFI and FADI scores, is less surprising given the equivocal findings regarding oral anti‐inflammatory medications compared to sham treatments for this condition [56]. Although, any observed benefits of heel lifts may have been overestimated by 17 participants (out of 35) having their treatment terminated in the Indomethacin group after three weeks. Finally, there was a large effect favoring custom orthotics when compared to heel lifts for reducing pain severity in calcaneal apophysitis, presenting an interesting contrast to the lack of difference observed when comparing heel lifts to prefabricated orthotics for the same condition. Differences in the materials (soft vs. hard) and processes for supply (custom vs. prefabricated) leading to non‐intervention effects are plausible explanations for the discrepancy in outcomes; however, further investigation is required to elucidate these findings.

An important consideration when interpreting the findings of this review relates to the quality of the trials that investigated the effectiveness of heel lifts. Using GRADE, the certainty of evidence of the findings reported in these trials was judged to be very low (39%), low (45%) and moderate (16%), which means we have limited confidence in the estimates of effect and they are likely to change when future trials are conducted [39]. Importantly, half of the outcomes (15/31) were at high risk of bias, mainly due to an absence of measures to blind the investigator, participant and/or both. Although we acknowledge the inherent difficulty in blinding participants to any physical interventions of any kind in clinical trials [57], performance bias is a risk across the included trials.

## 9 CLINICAL RESEARCH IMPLICATIONS

Comparisons were made between heel lifts and various comparator interventions, including eccentric calf exercise, ultrasound, stretching, prefabricated and custom orthotics, new shoes, education regarding activity modification, felt pads and analgesic medication. However, no trial compared heel lifts to a no treatment control (placebo, wait‐and‐see or sham), which is an understandable omission if the aim was to assess superiority between treatments instead of efficacy. However, the absence of no treatment‐based trials leaves uncertainty as to whether any observed effects of heel lifts are due to specific treatment effects, non‐specific factors such as placebo, the natural progression of the condition(s) or expectancy effects [58, 59]. Further high‐quality randomised controlled trials (comparing heel lifts to a no treatment control) are required to determine the efficacy of heel lifts for lower limb musculoskeletal conditions for which they are recommended.

## 10 STRENGTHS AND LIMITATIONS

The strengths of this review include the inclusion of only randomised trials and an appraisal of the evidence using RoB2 and GRADE, which are both recommended tools. This was performed by two independent people to reduce the risk of assessment bias. However, there are limitations requiring acknowledgment. First, analyses were from single trials as we were unable to perform a meta‐analysis due to the significant heterogeneity, which lowers our confidence in the estimates of effect. Second, this review omitted non‐English‐language trials. Systematic bias is unlikely to have been introduced by the English language restriction, but inclusion of more studies may have improved precision [60].

## 11 CONCLUSION

This systematic review of eight trials demonstrates that the current evidence for the efficacy and safety of heel lifts for lower limb musculoskeletal conditions is limited to mid‐portion Achilles tendinopathy, calcaneal apophysitis and plantar heel pain. There is very low certainty evidence for the benefit of heel lifts compared to indomethacin (analgesic medication) for plantar heel pain at 12 months; but not calcaneal apophysitis when compared to custom orthotics (low certainty evidence) at 12 weeks. The remaining (45 out of 47) outcomes of various comparators including eccentric calf exercise, ultrasound, cryotherapy, stretching, prefabricated orthotics, new shoes, activity modification education and felt pads found no clinically important differences between groups for the conditions assessed. Most of the evidence these findings are drawn from is of very low to low certainty, so there is a distinct possibility that future trials of high quality may change some of the findings of this review. Rigorous trials are needed to assess the clinical efficacy and safety of heel lifts for conditions for which they are currently recommended.

## AUTHOR CONTRIBUTIONS

**Jaryd Bourke**: Conceptualization; methodology; data curation; formal analysis; writing—original draft; writing—review and editing. **Shannon Munteanu**: Conceptualization; methodology; formal analysis; writing—review and editing; supervision. **Eman Merza**: Formal analysis; writing—review and editing. **Alessandro Garofolini**: Conceptualization; writing—review and editing; supervision. **Simon Taylor**: Conceptualization; writing—review and editing; supervision. **Peter Malliaras**: Conceptualization; methodology; formal analysis; writing—review and editing; supervision.

## CONFLICT OF INTEREST STATEMENT

The authors declare that they have no competing interests.

## ETHICS STATEMENT

Not applicable.

## CONSENT TO PARTICIPATE

Not applicable.

## SYSTEMATIC REVIEW PROTOCOL

A protocol was not prepared.

## CONSENT FOR PUBLICATION

Not applicable.

## Supporting information

Supporting Information S1

Supporting Information S2

Supporting Information S3

Supporting Information S4

Supporting Information S5

## Data Availability

The data presented in this review are included in the article and supplementary data files. Any additional data will be shared on reasonable request to the corresponding author (JB).
