# Supplementary material for: Efficacy of heel lifts for lower limb musculoskeletal conditions: A systematic review
Source: J Foot Ankle Res. 2024 Jun 15;17(2):e12031. doi: 10.1002/jfa2.12031 (PMC11296721; doi:10.1002/jfa2.12031)
Supplement: Supplementary file 1 — Supporting Information S1 [file JFA2-17-e12031-s004.docx]

| **Search term** | **MEDLINE** | **AMED** | **EMCARE** | **CINAHL** | **SPORTDiscus** |
| --- | --- | --- | --- | --- | --- |
| 1. injur* OR tend* OR fasc* OR bone* OR ligament* | 3,100,094 | 53,671 | 985,357 | 636,591 | 253,688 |
| 1. pain* OR disabilit* OR participat* | 2,009,984 | 79,810 | 967,925 | 825,713 | 238,146 |
| 1. S1 OR S2 | 4,802,104 | 118,891 | 1,786,395 | 1,355,475 | 443,761 |
| 1. OVID: heel ADJ2 (lift* OR rais* OR pad* OR elevat* OR ortho* OR cushion* OR insert*)  CINAHL: heel N2 (lift* OR rais* OR pad* OR elevat* OR ortho* OR cushion* OR insert*) | 1,187 | 291 | 784 | 632 | 623 |
| 1. S3 AND S4 | 602 | 179 | 316 | 402 | 354 |

**Additional File 1:** search strategy

**Limit S5 to humans Ovid MEDLINE and EMCARE*
