# Supplementary material for: Efficacy of heel lifts for lower limb musculoskeletal conditions: A systematic review
Source: J Foot Ankle Res. 2024 Jun 15;17(2):e12031. doi: 10.1002/jfa2.12031 (PMC11296721; doi:10.1002/jfa2.12031)
Supplement: Supplementary file 2 — Supporting Information S2 [file JFA2-17-e12031-s003.docx]

**Additional File 2:** hierarchy of outcomes

The outcomes of pain, function and disability, participant global assessment of treatment success, quality of life, participation and number of adverse events were extracted from trials where possible. Definitions of the patient-reported outcome measures are below, which were based on the ICON statement [1]. Where a trial reports multiple measures to evaluate the same outcome, we will extract only one outcome measure based on the following hierarchies, which were derived from previous reviews [2,3] and consensus between authors (JB, PM, SM).

**Pain**

Participant reported intensity of pain on performing a task/activity that loads the injured structure, or the pain intensity over a specific period (e.g., morning, night, 24 hours, a week, etc.). Pain can be measured on a numerical, categorical, or visual analogue scale, or a subscale of a composite measure, such as the Foot Function Index. If pain subscales of composite measures were not presented, we did not use total scores. We extracted outcomes in the following order:

1. worst pain over a specified time;
2. mean pain over a specified time;
3. worst pain with activity or load;
4. mean pain with activity or load;
5. unspecified pain;
6. daytime pain;
7. rest pain;
8. night-time pain;
9. Other scores as reported by trials.

**Function and disability**

Participants reporting their ability or inability to perform activities/tasks (e.g., activities of daily living, work, etc.) due to pain. Function and disability can be measured using specific disability or function subscales in measures such as the Foot Function Index, Foot Ankle Disability Index, Oxford Foot Ankle Questionnaire, or other validated measures. If function and disability subscales of composite measures were not presented, we did not use total scores. We extracted outcomes in the following order:

1. Lower Extremity Functional Scale (LEFS) [4];
2. Foot and Ankle Ability Measure (FAAM) [5];
3. Foot Function Index (FFI) [6];
4. Foot and Ankle Disability Index (FADI) [7];
5. Oxford Foot and Ankle Questionnaire (OxFAQ) [8];
6. Other scores as reported by trials.

**Participant rating of overall condition**

A single numerical evaluation of change (e.g., how are you now compared with prior to the treatment?) measured using a Global Rating of Change scale [9].

**Participation**

Participant rating of the level of participation/engagement across areas of their life (e.g., ratings of level of sport and time to return to sport). If participation subscales of composite measures were not presented, we did not use total scores. We extracted outcomes in the following order:

1. Objective measures of physical activity (e.g., accelerometer) [10];
2. 7-day Recall Physical Activity Questionnaire [11];
3. International Physical Activity Questionnaire [12];
4. Other scores as reported by trials.

**Quality of life**

The general wellbeing of the individual measured using specific questionnaires (e.g., European QoL-5 dimension [13], Short-Form 12 [14], etc.,).

**Composite measures**

Composite measures that encompass multiple domains (e.g., pain, disability, etc.) are common in trials assessing non-surgical interventions for musculoskeletal conditions. Therefore, any composite measure that encompassed one or more domains of pain, function and disability, participant rating overall condition, quality of life and participation will be extracted and reported.

**Adverse events**

The number of any adverse events, such as new pain, to any interventions used in the trial.

**References**

1. Vicenzino B, de Vos R, Alfredson H, et al. ICON 2019—International Scientific Tendinopathy Symposium Consensus: There are nine core health-related domains for tendinopathy (CORE DOMAINS): Delphi study of healthcare professionals and patients. *Br J Sports Med* 2020;54:444-51. doi:10.1136/bjsports-2019-100894.
2. Karjalainen T, Jain N, Page C, et al. Subacromial decompression surgery for rotator cuff disease. *Cochrane Database Syst Rev*2019;1:CD005619. doi:10.1002/14651858.CD005619.pub3.
3. David J, Sankarapandian V, Christopher P, et al. Injected corticosteroids for treating plantar heel pain in adults. *Cochrane Database Syst Rev* 2017;6:CD009348. doi:10.1002/14651858.CD009348.pub2.
4. Binkley J, Stratford P, Lott S, et al. The Lower Extremity Functional Scale (LEFS): scale development, measurement properties, and clinical application. North American Orthopaedic Rehabilitation Research Network. *Phys Ther* 1999;79(4):371-83.
5. Martin R, Irrgang J, Burdett R, et al. Evidence of validity for the Foot and Ankle Ability Measure (FAAM). *Foot Ankle Int* 2005;26:968-83. doi:10.1177/107110070502601113.
6. Budiman-Mak E, Conrad K, Roach K. The Foot Function Index: a measure of foot pain and disability. *J Clin Epidemiol* 1991;44(6):561-70. doi:10.1016/0895-4356(91)90220-4.
7. Martin R, Burdett R, Irrgang J. Development of the Foot and Ankle Disability Index (FADI). *J Orthop Sports Phys Ther* 1999;29:32-3.
8. Morris C, Doll H, Wainwright A, et al. The Oxford ankle foot questionnaire for children: scaling, reliability and validity. *J Bone Joint Surg Br* 2008;90:1451-6. doi:10.1302/0301-620X.90B11.21000.
9. Kamper S, Maher C, Mackay G. Global rating of change scales: a review of strengths and weaknesses and considerations for design. *J Man Manip Ther* 2009;17(3):163-70. doi:10.1179/jmt.2009.17.3.163.
10. Quinlan C, Rattray B, Pryor D, et al. The accuracy of self-reported physical activity questionnaires varies with sex and body mass index. *PLoS One* 2021;16(8):e0256008. doi:10.1371/journal.pone.0256008.
11. Sallis J, Haskell W, Wood P, et al. Physical activity assessment methodology in the five- city project. *Am J Epidemiol* 1985;121:91–106. doi:10.1093/oxfordjournals.aje.a113987.
12. Craig C, Marshall A, Sjöström M, et al. International physical activity questionnaire: 12-country reliability and validity. *Med Sci Sports Exerc* 2003;35(8):1381-95. doi:10.1249/01.MSS.0000078924.61453.FB.
13. EuroQoL Group. EuroQoL: a new facility for the measurement of heath-related quality of life. *Health Policy* 1990;16(3):199–208.
14. Ware J, Kosinski M, Keller S. A 12-Item Short-Form Health Survey: construction of scales and preliminary tests of reliability and validity. *Med Care* 1996;34(3):220-33. doi:10.1097/00005650-199603000-00003.
