# Supplementary material for: Efficacy of heel lifts for lower limb musculoskeletal conditions: A systematic review
Source: J Foot Ankle Res. 2024 Jun 15;17(2):e12031. doi: 10.1002/jfa2.12031 (PMC11296721; doi:10.1002/jfa2.12031)
Supplement: Supplementary file 3 — Supporting Information S3 [file JFA2-17-e12031-s002.docx]

**Additional File 3:** Rules for GRADE assessment

The certainty of evidence was graded as high, moderate, low, or very low for each of the following: risk of bias, indirectness, and imprecision. The outcomes for each comparison were classified into four categories: (1) high (further research is very unlikely to change our confidence in the estimate of effect), (2) moderate (further research is likely to have an important impact on our confidence in the estimate of effect and may change the estimate), (3) low (further research is very likely to have an important impact on our confidence in the estimate of effect and is likely to change the estimate) and (4) very low (we are very uncertain about the estimate) [1]. Our rules for determining each outcomes grade were derived from the GRADE [2] and Cochrane handbook [3,4], and consensus among authors (JB, PM, SM).

**Risk of bias**

Bias judgements were derived from the Risk of Bias 2.0 scores at each outcome. The outcomes grade was downgraded by one if they had some concerns of risk of bias (that would likely lower the confidence in the estimate of effect) and by two if there was a very serious risk of bias (at least one criterion graded as high risk of bias in the Risk of Bias 2.0). Outcomes were not downgraded if there was a low risk of bias or some concerns of risk of bias that would unlikely lower the confidence in the estimate of effect [5].

**Inconsistency**

Inconsistency is only applicable where there is meta-analysis, which was not possible for our review [6]. Therefore, no outcomes grade was downgraded for inconsistency.

**Indirectness**

Indirectness was assessed based on the following questions:

1. is the cohort representative of the intended population;
2. is the setting or context transferable;
3. are the interventions clinically relevant;
4. are the outcomes important.

The outcomes grade was downgraded by one per criteria not satisfied to maximum possible downgrade of two.

**Imprecision**

For continuous data we calculated the optimal information size (OIS), using a pooled standard deviation (from the trial), 80% power, alpha < 5%, and minimal important difference (MID) specific to the outcome (where possible) described below:

| Outcome | MID |
| --- | --- |
| Visual analogue scale | 8 mm [7] |
| VISA-A | 14 points [8] |
| FFI | 12 points [9] |
| Face pain scale - revised | 2 points [10] |

If we were unable to identify a suitable minimal important difference, we used 10% of the maximum possible score of the outcome. If an outcomes sample size did not meet or exceed the OIS (allowing for 10% as a leniency measure), we downgraded that outcome by one. If the outcome was not reported in sufficient detail to calculate a pooled standard deviation, we downgraded that outcome by one. If an outcome satisfied the OIS but had a wide confidence interval spanning between no difference to greater than the minimal important difference the outcome was downgraded by one.

Outcomes that satisfied the OIS and did not have a wide confidence interval that spanned between no difference to greater than the minimal important difference were not downgraded. We did not downgrade the outcome measure 7-day physical activity recall, as there is no MID listed in the literature and no possible maximum score. For dichotomous data (i.e., adverse events), we applied the same rule and assumed a relative risk difference of 25% as the minimal clinically important difference.

**Publication bias**

The possibility of publication bias was not assessed as there was an insufficient number of included studies (10 or less) to construct a funnel plot [11]. Therefore, no outcome was downgraded for publication bias.

**References**

1. Balshem H, Helfand M, Schünemann HJ, et al. GRADE guidelines: 3. Rating the quality of evidence. *J Clin Epidemiol* 2011;64(4):401–6. doi:10.1016/j.jclinepi.2010.07.015.
2. Schünemann H, Brożek J, Guyatt G, Oxman A. GRADE handbook for grading quality of evidence and strength of recommendations. Updated October 2013. The GRADE Working Group, 2013. Available from guidelinedevelopment.org/handbook. Accessed August 2023.
3. Schünemann H, Higgins J, Vist G, Glasziou P, Akl E, Skoetz N, Guyatt G. Chapter 14: Completing ‘Summary of findings’ tables and grading the certainty of the evidence. In: Higgins J, Thomas J, Chandler J, Cumpston M, Li T, Page M, Welch V, ed. *Cochrane Handbook for Systematic Reviews of Interventions* version 6.4 (updated August 2023). Cochrane, 2023.
4. Schünemann H, Vist G, Higgins J, Santesso N, Deeks J, Glasziou P, Akl E, Guyatt G. Chapter 15: Interpreting results and drawing conclusions. In: Higgins J, Thomas J, Chandler J, Cumpston M, Li T, Page M, Welch V, ed. *Cochrane Handbook for Systematic Reviews of Interventions* version 6.4 (updated August 2023). Cochrane, 2023.
5. Higgins J, Savović J, Page M, Elbers R, Sterne J. Chapter 8: Assessing risk of bias in a randomized trial. In: Higgins J, Thomas J, Chandler J, Cumpston M, Li T, Page M, Welch V, ed. *Cochrane Handbook for Systematic Reviews of Interventions* version 6.4 (updated August 2023). Cochrane, 2023.
6. Guyatt G, Oxman A, Kunz R, et al. GRADE working group. GRADE guidelines: 7. Rating the quality of evidence--inconsistency. *J Clin Epidemiol* 2011;64(12):1294-302. doi:10.1016/j.jclinepi.2011.03.017.
7. Landorf K, Radford J, Hudson S. Minimal Important Difference (MID) of two commonly used outcome measures for foot problems. *J Foot Ankle Res* 2010;3:7. doi:10.1186/1757-1146-3-7.
8. Lagas I, van der Vlist A, van Oosterom R, et al. Victorian Institute of Sport Assessment-Achilles (VISA-A) questionnaire-minimal clinically important difference for active people with midportion Achilles tendinopathy: a prospective cohort study. *J Orthop Sports Phys Ther* 2021;51(10):510-16. doi:10.2519/jospt.2021.10040.
9. Landorf K, Radford J. Minimal important difference: Values for the Foot Health Status Questionnaire, Foot Function Index and Visual Analogue Scale. *The Foot* 2008;18:15-19. doi:10.1016/j.foot.2007.06.006.
10. Tsze D, Hirschfeld G, von Baeyer C, et al. Clinically significant differences in acute pain measured on self-report pain scales in children. *Acad Emerg Med* 2015;22(4):415-22. doi:10.1111/acem.12620.
11. Egger M, Davey Smith G, Schneider M, et al. Bias in meta-analysis detected by a simple, graphical test. *BMJ* 1997;315(7109):629-34. doi:10.1136/bmj.315.7109.629.
